# Supplementary material for: Nurse’s spiritual care competence in Ethiopia: A multicenter cross-sectional study
Source: PLoS One. 2022 Mar 10;17(3):e0265205. doi: 10.1371/journal.pone.0265205 (PMC8912899; doi:10.1371/journal.pone.0265205)
Supplement: S1 File — (DOCX) [file pone.0265205.s001.docx]

## Annex I: English version Questionnaire

**General Instructions**

- You are not expected to write your name.
- Put tick mark for your answer for your background.
- Encircle the letter of your answer for choice items.
- Write your answer of open-ended questions on the space provided after the questions.

This question is intended only for academic purpose and the researcher assure you that your response would be kept confidential.

**Part I: Socio-demographic factors**

1. Age____________
2. Sex A. Male B. Female
3. Religion A. Orthodox B. Muslim C. Protestant D. Other
4. Marital status A. Single B. Married C. Divorced D. Widowed
5. Educational status A. Diploma B. Bachelor degree C. MSc

**Part II: Work related factors**

1. Clinical experience in year___________
2. Current organizational Position A. Staff nurse B. Head nurse C. Supervisor nurse
3. Employment Type A. Formal B. Contractual C. Other
4. Type of ward you are currently working ___________________
5. Have you ever received any training for spiritual care? A. Yes B. No

**Part III: Spiritual Care Competence Scale (SCCS)**

For each item, please estimate your own level of competency by **mark’˅’** an answer which best reﬂects the extent to which you agree or disagree with each statement.

| S.no |  | Strongly agree | Agree | Neutral | Disagree | Strongly disagree |
| --- | --- | --- | --- | --- | --- | --- |
|  | **Assessment and implementation of spiritual care** |  |  |  |  |  |
| 1 | I can report orally and/or in writing on a patient’s spiritual needs |  |  |  |  |  |
| 2 | I can tailor care to a patient’s spiritual needs/problems in consultation with the patient |  |  |  |  |  |
| 3 | I can tailor care to a patient’s spiritual needs/problems through multidisciplinary consultation |  |  |  |  |  |
| 4 | I can record the nursing component of a patient’s spiritual care in the nursing plan |  |  |  |  |  |
| 5 | I can report in writing on a patient’s spiritual functioning |  |  |  |  |  |
| 6 | I can report orally on a patient’s spiritual functioning |  |  |  |  |  |
|  | **Professionalization and improving the quality of spiritual care** |  |  |  |  |  |
| 7 | Within the nursing ward, I can contribute to quality assurance in the area of spiritual care |  |  |  |  |  |
| 8 | Within the nursing ward, I can contribut to professional development in the area of spiritual care |  |  |  |  |  |
| 9 | Within the nursing ward, I can identify problems relating to spiritual care in peer discussion sessions |  |  |  |  |  |
| 10 | I can coach other care workers in the area of spiritual care delivery to patients |  |  |  |  |  |
| 11 | I can make policy recommendations on aspects of spiritual care to the management of the nursing ward |  |  |  |  |  |
| 12 | I can implement a spiritual care improvement project in the nursing ward |  |  |  |  |  |
|  | **Personal support and patient counseling** |  |  |  |  |  |
| 13 | I can provide a patient with spiritual care |  |  |  |  |  |
| 14 | I can evaluate the spiritual care that I have provided in consultation with the patient and in the disciplinary/multidisciplinary team |  |  |  |  |  |
| 15 | I can give a patient information about spiritual facilities within the care institution (including spiritual care, meditation center, religious services) |  |  |  |  |  |
| 16 | I can help a patient continue his or her daily spiritual practices (including providing opportunities for rituals, prayer, meditation, reading the Bible/Koran, listening to music) |  |  |  |  |  |
| 17 | I can attend to a patient’s spirituality during the daily care (e.g. physical care) |  |  |  |  |  |
| 18 | I can refer members of a patient’s family to a spiritual advisor/pastor, etc. if they ask me and/or if they express spiritual needs |  |  |  |  |  |
|  | **Referral** |  |  |  |  |  |
| 19 | I can effectively assign care for a patient’s spiritual needs to another care provider/care worker/care discipline |  |  |  |  |  |
| 20 | At the request of a patient with spiritual needs, I can in a timely and effective manner refer him or her to another care worker (e.g. a chaplain/the patient’s own priest/imam) |  |  |  |  |  |
| 21 | I know when I should consult a spiritual advisor concerning a patient’s spiritual care |  |  |  |  |  |
|  | **Attitude towards patient spirituality** |  |  |  |  |  |
| 22 | I show unprejudiced respect for a patient’s spiritual/religious beliefs regardless of his or her spiritual/religious background |  |  |  |  |  |
| 23 | I am open to a patient’s spiritual/religious beliefs, even if they differ from my own |  |  |  |  |  |
| 24 | I do not try to impose my own spiritual/religious beliefs on a patient |  |  |  |  |  |
| 25 | I am aware of my personal limitations when dealing with a patient’s spiritual/religious beliefs |  |  |  |  |  |
|  | **Communication** |  |  |  |  |  |
| 26 | I can listen actively to a patient’s ‘life story’ in relation to his or her illness/handicap |  |  |  |  |  |
| 27 | I have an accepting attitude in my dealings with a patient (concerned, sympathetic, inspiring trust and conﬁdence, empathetic, genuine, sensitive, sincere and personal) |  |  |  |  |  |
