# Supplementary material for: Nurse’s spiritual care competence in Ethiopia: A multicenter cross-sectional study
Source: PLoS One. 2022 Mar 10;17(3):e0265205. doi: 10.1371/journal.pone.0265205 (PMC8912899; doi:10.1371/journal.pone.0265205)
Supplement: Attachment — Submitted filename: Author response 2.docx [file pone.0265205.s003.docx]

Author’s response to reviews

Title: Nurse’s spiritual care competence in Ethiopia: a multicenter cross-sectional study

Authors:

Kalid Seid (Kalidseid7@gmail.com)

Adem Abdo (ademabdo448@gmail.com)

Version: 2 Date: 22 February 2022

Author’s response to reviews:

General response:

Thank you for giving us the opportunity to submit a revised draft of our manuscript titled “Nurse’s spiritual care competence in Ethiopia: a multicenter cross-sectional study” to [*PLOS ONE*]. We appreciate the time and effort that editors and reviewers have dedicated to providing your valuable feedback on our manuscript. We are grateful to the editors and reviewers for their insightful comments on our paper. We have been able to incorporate changes to reflect most of the suggestions provided by the editors and reviewers. We hope that the revised manuscript has now addresses all of the editors and reviewers comments. Please find attached the point-by-point response to those comments as well as the revised manuscript with track changes as well as the clean copy manuscript. In preparing the manuscript, we have strictly followed journal instructions for authors. Looking forward to hearing from you soon.

Here is a point-by-point response to the editor’s comments and concerns.

**Comments from Editor**

**Comment 1:** *[Please review your reference list to ensure that it is complete and correct. If you have cited papers that have been retracted, please include the rationale for doing so in the manuscript text, or remove these references and replace them with relevant current references. Any changes to the reference list should be mentioned in the rebuttal letter that accompanies your revised manuscript. If you need to cite a retracted article, indicate the article’s retracted status in the References list and also include a citation and full reference for the retraction notice.

Additional Editor Comments: Add any references that the reviewer has indicated]*

**Response:** Thank you for your valuable feedback. We have thoroughly checked all references and we didn’t get retracted paper. We added reference number 6 to address the impact of spirituality on chronic illness patients that raised by the reviewer. We highlighted the change within the track change and you can also see from clean copy of revised version.
